# A malaria seasonality dataset for sub-Saharan Africa

**DOI:** 10.1038/s41597-025-05996-5

**Published:** 2025-10-28

**Authors:** Francesca Sanna, Suzanne H. Keddie, Tara Boyhan, Paulina A. Dzianach, Michael McPhail, Julia Seitz, Thomas Nguyen, Adrian Redpath, Twatasha Chikolwa, Annie J. Browne, Jailos Lubinda, Adam Saddler, Sarah Hafsia, Rubi Jayaseelen, Hunter S. Baggen, Jennifer A. Rozier, Tasmin L. Symons, Joseph Harris, Sarah Connor, Camilo Vargas, Charles Whittaker, Michele Nguyen, Peter W. Gething, Daniel J. Weiss

**Affiliations:** 1 Malaria Atlas Project, The Kids Research Institute Australia, Nedlands, WA 6009 Australia; 2 St John’s Institute of Dermatology, King’s College London, London, UK; 3 School of Population Health, Curtin University, Bentley, WA 6102 Australia; 4 MRC Centre for Global Infectious Disease Analysis, Imperial College London, London, UK; 5 Division of Infectious Diseases & Vaccinology, School of Public Health, University of California, Berkeley, USA; 6 Lee Kong Chian School of Medicine, Nanyang Technological University, Singapore, Singapore; 7 School of Physical & Mathematical Sciences, Nanyang Technological University, Singapore, Singapore

**Keywords:** Malaria, Climate sciences

## Abstract

Malaria imposes a significant global health burden and remains a major cause of child mortality in sub-Saharan Africa. In many countries, malaria transmission varies seasonally. The use of seasonally-deployed interventions is expanding, and the effectiveness of these control measures hinges on quantitative and geographically-specific characterisations of malaria seasonality. Malariometric timeseries from routine surveillance data and scientific and programmatic literature offer a resource for modelling patterns of malaria seasonality. This study creates and makes publicly available a geolocated dataset of historical timeseries describing malaria seasonality published since 2000 for sub-Saharan Africa. We used three approaches to assemble the dataset: i) an extensive literature review that included novel natural language processing to accelerate screening of published articles, ii) extractions from a routine surveillance dataset that contains geolocated data from all malaria-endemic countries, and iii) cross-referencing and incorporation of timeseries from a key entomological dataset. The resulting data include malaria prevalence, incidence, mortality, and entomological timeseries; and a novel assembly of qualitative descriptions of malaria seasonality extracted from published literature.

## Background & Summary

Despite massive efforts made over the past decades, malaria remains a substantial public health issue globally. Sub-Saharan African countries shoulder the heaviest burden of disease, accounting for the majority of global mortality and morbidity, especially in children aged under five^1^. *Plasmodium falciparum* is the most prevalent and virulent malaria species in Africa and was responsible for an estimated 246 million malaria cases in the WHO African Region in 2023^1^.

In many parts of sub-Saharan Africa, malaria transmission displays seasonal variation. The largest driver of seasonality is rainfall, although non-rainfall climatic drivers such as temperature and humidity also play important roles^2,3^. Further factors influencing seasonality include socioeconomic determinants and human population movement^4–8^. Likewise, seasonally-deployed interventions such as seasonal malaria chemoprevention^9^, insecticide treated mosquito nets^8^, and indoor residual spraying^10,11^ that can suppress malaria transmission, thereby altering the resulting seasonal profile of the disease.

Numerous high-resolution datasets have been developed to characterize the environmental conditions that are hypothesized to drive seasonal patterns in malaria^12^, but comparably rich datasets of malaria timeseries, while often recorded, have not been systematically assembled and shared publicly. This hampers efforts to robustly model seasonal malaria patterns across a wide range of climatological and sociodemographic settings.

Significant challenges are associated with developing datasets of malaria timeseries from the published literature. First, studies containing timeseries data on relevant malaria metrics are dispersed across a wide range of topics, and are often unrelated to seasonality. As a result, targeted keyword searches are insufficiently sensitive to generate comprehensive search results. Second, the geographical units being reported on vary greatly and timeseries may represent anything from a single point location to an entire country. Third, the malaria metrics being reported on are also highly variable, making inter-metric comparison complex. Finally, timeseries data are often presented in papers only via a plotted graphic, rather than as tabular values.

This study presents the outcome of an extensive exercise to build an unprecedentedly rich and comprehensive assembly of published malaria timeseries in sub-Saharan Africa. The dataset was built via i) an extensive literature review that included novel natural language processing to accelerate screening of published articles, ii) extractions from a routine surveillance database that contains geolocated data such as malaria parasite incidence data from malaria-endemic countries, and iii) cross-referencing and incorporation of timeseries from key pre-existing datasets. The resulting dataset consists of malaria prevalence, incidence, entomological, and mortality timeseries, as well as anecdotal information that constitute localised expert opinions of malaria seasonal patterns found in peer-reviewed publications.

## Methods

### Dataset structure

The malaria seasonality data are stored in an instance of a relational database system that we export in tabular form for use by researchers and make publicly available at 10.6084/m9.figshare.28879805. Each row in the seasonality dataset consists of a single malaria seasonality record for a given time and place. Columns in the dataset are described in the Supplemental Information and can be grouped into the following categories: (1) geographic location, (2) dates of data collection, (3) the malaria metric captured, (4) the source of the data (e.g., publication citation), and (5) the observed values at a monthly resolution. To maximise flexibility for future analyses, the dataset was designed to include any data that could contain information relevant to analyses of malaria seasonality, including anecdotal mentions of seasonal timing published in peer-reviewed journals. To bridge anecdotal and empirical data, each row also contains a simplified monthly timeseries with ‘peak’ months codified with a 1, second peak (if present) with a 0.5, and other months codified with a zero. For anecdotal data, the peak months were taken directly from the text, while the simplified summaries derived from the empirical data were processed by assigning the highest value from the timeseries as the peak. Contiguous months exceeding one standard deviation (SD) from the monthly mean value were then used to define the extent of each peak period. Two further rules where applied. First, when there was a fragmented peak (such as peak spanning three months with the middle month not exceeding 1 * SD) the peak was considered to include the apparent gap. Second, in the absence of monthly values exceeding 1 * SD from the mean, peaks were still identified if the timeseries exhibited a continuous period of at least three months with values exceeding 0.5 * SD over the mean. We used this approach to define “in season” as there is no universally agreed upon definition for this metric. Our choice was guided by the need for a measure that was independent of scale as we were attempting to compare data for varied metrics and from areas with vastly different burdens of malaria. The use of standard deviation satisfies the requirement of scale independence. We also needed an approach that could distinguish between places with seasons and places without seasons, which ruled out simpler definitions of peak such as observing more than 1/12^th^ of the annual cases in a month. Lastly, the peak months defined by the anecdotes aligned strongly with geographically coincident measured malaria timeseries in terms of the timing of the defined peaks. However, the anecdotal peaks tended to be longer than the peaks derived from the observed timeseries. We attributed the narrower peaks among the data rows to greater geographic specificity, as a much higher proportion of the data rows were from measurements collected at point locations. In contrast, the anecdotal rows were frequently attributed to national or the largest subnational units. Also relevant for explaining the longer peak lengths among the anecdotal data is interannual variability, with anecdotes generalised across many years and thus including transmission seasons starting early, lasting longer than normal, etc.

### Data gathering

#### Literature review and data extraction

We carried out a literature review of papers published from January 2000 to December 2022, using the following broad keyword search “malaria AND *list of any endemic Country in sub-Saharan Africa”*. Due to the large number of results returned from the PubMed query, we developed a seasonality literature review classifier (SLRC) that utilised natural language processing, a form of machine learning, to identify potentially relevant sources from our initial PubMed search results. The SLRC was initially trained using the results of an ad-hoc malaria seasonality literature review, and it was iteratively trained thereafter using the results of the manual abstract classification process (see section *Technical Validation* for further details). The iterative nature of this training process allowed us to continuously retrain and improve the accuracy of the SLRC model over time. Researchers manually screened all abstracts deemed to be relevant by the SLRC and a full-text review was conducted for all papers with abstracts that had passed both the natural language processing and manual assessment. The full-text review process applied the inclusion/exclusion criteria of our study protocol (see Supplemental Information), and all malaria timeseries were extracted from the text, figures, or tables, for entry into our dataset.

#### Routine surveillance dataset

The Malaria Atlas Project (MAP) has curated an extensive, annually updated dataset of geolocated clinical case incidence and mortality data collected from all malaria-endemic countries. The dataset contains over 340,000 national and subnational values from 1980 to 2023 from relevant sources that have been systematically identified, assessed for quality and consistency, and ingested into the routine surveillance dataset. Most of these routine data have an annual temporal resolution, and only 236 timeseries from sub-Saharan African countries contained weekly or monthly timeseries for a complete year and were thus included in the seasonality dataset. Only publicly available routine surveillance data were included, and the data source for each malaria timeseries is identified in the dataset. We excluded any sources for which we did not have permission to publicly share the information.

#### Incorporating existing compiled data

We utilised the PANGAEA dataset (https://doi.pangaea.de/10.1594/PANGAEA.892682) dataset as a reference list for sources containing malaria seasonality timeseries. PANGAEA contains malaria entomological inoculation rate data at monthly temporal resolution, with durations of at least one year, from sub-Saharan Africa, and collected between 1968 to 2013^12^. To ensure consistency with our dataset, we reextracted the entomological inoculation rate timeseries using our protocols for the sources within PANGAEA. Because the PANGAEA timeseries predates our 2000–2022 collection, we extracted timeseries identified by PANGAEA only from 2000–2013.

#### Inclusion/exclusion criteria

The final dataset was created by employing the following study eligibility criteria: i) contains malaria seasonality information; ii) reported data were able to be geolocated to either a point location with an associated latitude and longitude, or to a national or subnational administrative unit polygon; iii) available timeseries span at least 12 consecutive months; and iv) if the full text for the study was obtainable. Additionally, we included anecdotal information on seasonal timing when the peak month or period was clearly defined in the text. All study designs from published papers were deemed eligible unless the timeseries measurements were collected as a component of a study quantifying the impact of an intervention regime change.

#### Data and anecdotal information

Malaria transmission in sub-Saharan Africa is often seasonal and can vary considerably between locations and years, even within the same country^2^. We developed this dataset to support analyses of this complex aspect of malaria epidemiology. The resulting dataset is the most comprehensive collection of data quantifying seasonal patterns of malaria in sub-Saharan Africa. A novel aspect of the dataset is that it contains both empirical data and anecdotal information from published articles and reports. The anecdotal information, which represents 14% of the rows in the dataset, was included to capture the knowledge of local experts when available in published reports, and to the best of our knowledge, this type of data has not previously been systematically recorded. By including both types of information, our dataset may support analyses exploring the differences between perceived and measured seasonal patterns.

The anecdotal data were potentially imprecise relative to the timeseries. As such, anecdotal data were included using the following protocol. First, we only included the information when it clearly stated the peak month or series of months for malaria transmission, malaria cases, or vector density (e.g., “The peak of malaria transmission in district X is between July and October”). Notably, we did not include tangential metrics such as “rainy season” unless they were directly linked to malaria within the same publication. Second, if a paper cited a reference with an anecdotal statement about malaria seasonality, we reextracted that information from the original source when it was available if it was suitable per our inclusion criteria. Because we recognise that anecdotal information may be biased, our dataset clearly defines which rows contain timeseries and which contain only anecdotal information. As such, researchers using the dataset can easily choose which type(s) of data they use for their analyses.

#### Dataset description

**Fig. 1:**
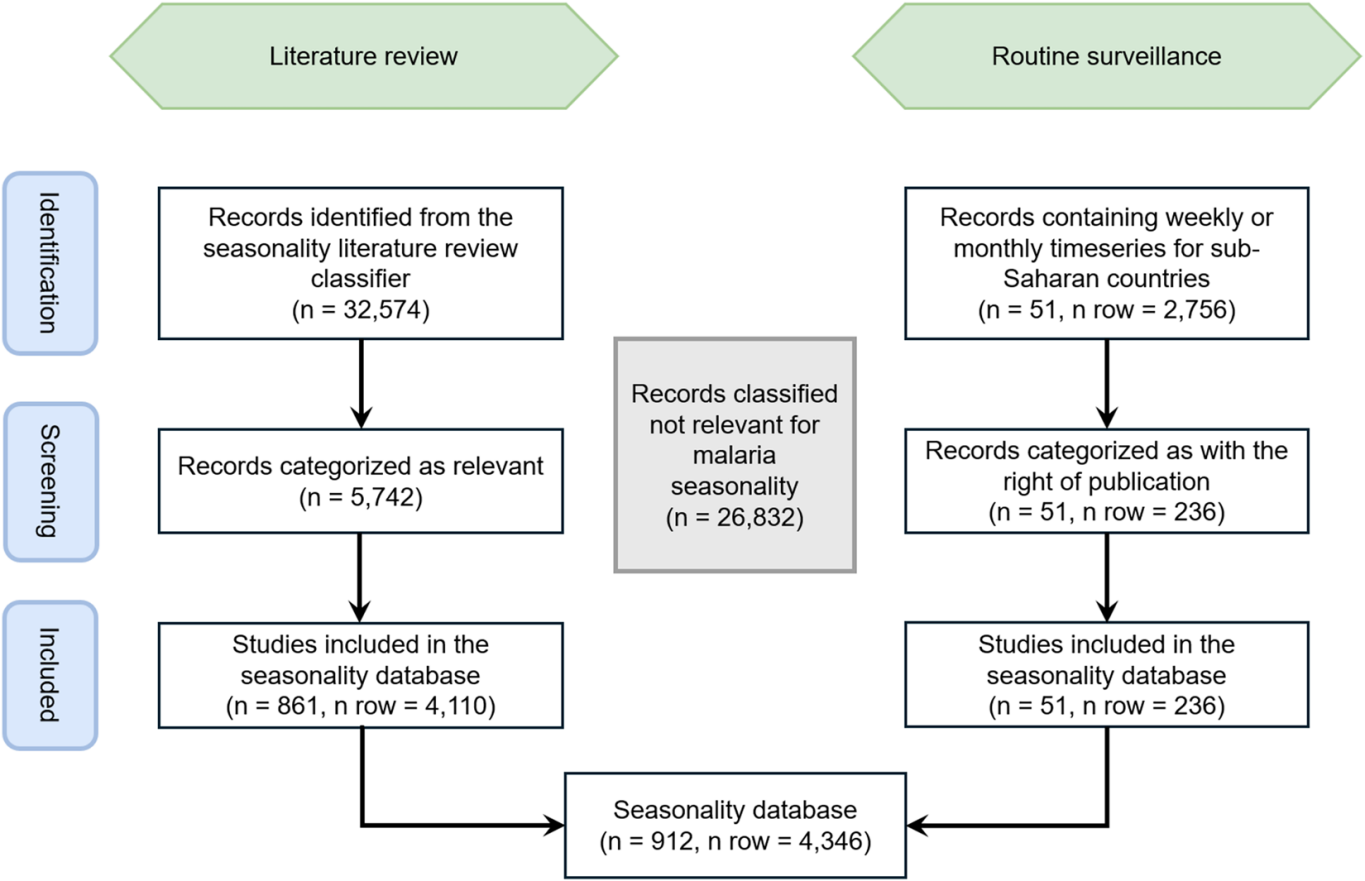
Flowchart of the data source identification and data review process. Within the figure, “n” signifies the number of sources while “n row” signifies the number of rows in the dataset, each containing a unique malaria seasonality timeseries or anecdote.

**Fig. 2:**
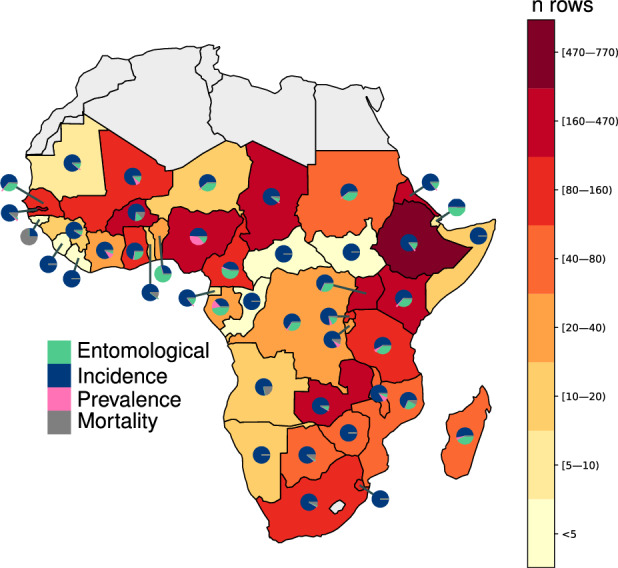
Geographical data coverage within the seasonality dataset. Each country is coloured according to the number of rows (i.e. unique annual timeseries) identified. Pie charts show the proportional breakdown by country between the categories of metric.

**Fig. 3:**
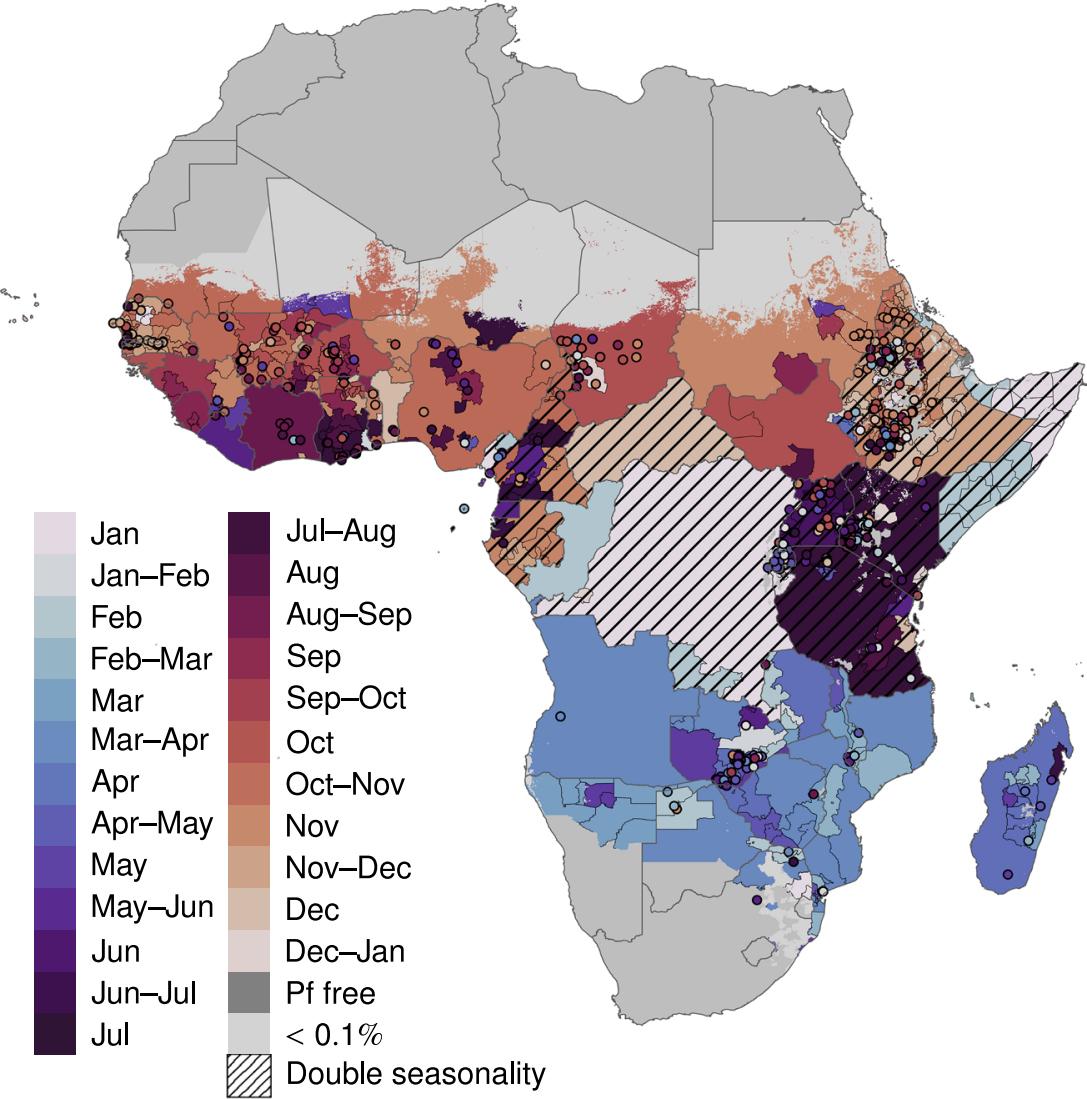
Map of seasonality data for clinical incidence. Available data for each geographical unit (country [ADMIN0], administrative levels 1-3, or point) are summarised by the mode peak timing across all geographically matched timeseries. Units with bimodal timeseries and/or anecdotal information indicating two seasonal peaks each year are indicated by cross-hatching.

The traditional literature review conducted using PubMed identified 32,574 potentially relevant peer-reviewed or pre-print articles that were published between 2000 and 2022. Some of these papers included data collected earlier, which resulted in extracted timeseries stretching from 1964 to 2021, but timeseries dating before 2000 were not gathered systematically and should be considered opportunistic inclusions to the dataset. The SLRC reduced this initial set to 5,742 sources it deemed relevant (Fig. 1) and thus eligible for full-text review. The full-text review yielded 790 new sources, to which we added 71 papers identified by the PANGAEA study that our literature review initially failed to capture. The 861 literature sources resulted in 4,110 unique records of malaria seasonality in sub-Saharan Africa. These were combined with 236 observations from MAP’s routine surveillance database to produce a final dataset containing 4,346 records (rows) (Table 1). The resulting dataset includes information on the source, the start and the end of the study period, the population study group (e.g., only females), and the location of the study. Full descriptions of the data columns are provided in the Supplemental Information. Location information is provided for each row at the lowest identifiable administrative level, or using latitude and longitude for points. Fourteen percent of the dataset consists of rows derived from anecdotal descriptions, while 86% of the rows were supported by data. Among the data rows, 78% contain a numerical timeseries, 5% are associated with plots from which numerical data are impossible to extract but we could ascertain peak periods, and 3% are associated with written descriptions of seasonal timings that were observed in unpublished data (i.e., the reported seasonality was based on data and therefore not considered anecdotal). The most common metrics are incidence (73%), entomological (20%), prevalence (4%), and mortality (2%). Fifty percent of the malaria seasonality observations are available at point level and have longitude and latitude attached, followed by the administrative levels two (23%), one (13%), zero (i.e., national) (8%), and three (6%) (Table 2). Information is available from 47 sub-Saharan African countries (Fig. 2), 43 of which have at least one timeseries available (Fig. 2). However, the density of data is asymmetrically distributed across the continent and seasonality may be poorly characterised in places with very few associated rows. Of particular concern are countries on or very near the Equator that lack regular peaks in malaria transmission. Figure 3 shows a map of the mode primary peak month for malaria incidence seasonality from sources within our dataset. To illustrate the heterogeneity present in the dataset, even within single countries, the map in Fig. 3 is displayed hierarchically such that higher resolution administrative units overlay lower-level ones (e.g., subnational units are on top of countries). Features of this map include a primary peak between September and October for the Sahel Region, and peaks between March and May for Southern Africa. This map should be interpreted cautiously, however, as it reflects data from only the primary peak (or period) of sampled locations within the dataset. As such, it may reflect inaccuracies related to small sample sizes and/or timeseries collected with localised epidemiological settings that differ from general trends within the country.

**Table 1:** Summary of data within the MAP seasonality dataset.

	Literature review	Routine surveillance dataset	Total
**Extracted**	**n**	**n**	**n**
Rows	4,110	236	4,346
Unique sources	861	51	912
**Type of data**	**n rows (%)**	**n rows (%)**	**n rows (%)**
Data	3,493 (85.0)	236 (100)	3,729 (85.8)
Anecdotal	617 (15.0)	N/A	617 (14.2)
**Timeseries available**	**n rows (%)**	**n rows (%)**	**n rows (%)**
Yes	3,136 (76.3)	236 (100)	3,372 (77.6)
No	755 (18.5)	N/A	755 (17.4)
Unable to extract	219 (5.3)	N/A	219 (5.0)
**Metric**	**n rows (%)**	**n rows (%)**	**n rows (%)**
Incidence	2,967 (72.2)	216 (91.5)	3,183 (73.3)
Entomological	875 (21.3)	N/A	875 (20.1)
Prevalence	187 (4.5)	N/A	187 (4.3)
Mortality	81 (2.0)	20 (8.5)	101 (2.3)
**Total**	**4,110**	**236**	**4,346**

**Table 2:** Summary of administrative unit and point level data within the seasonality dataset.

	Literature review	Routine surveillance dataset	Total	Total timeseries available
**Admin Level**	n rows (%)	n rows (%)	n rows (%)	n rows (%)
ADMIN0	313 (7.6)	99 (42.0)	412 (9.5)	271 (8.0)
ADMIN1	539 (13.1)	81 (34.3)	620 (14.3)	435 (12.9)
ADMIN2	899 (21.9)	56 (23.7)	955 (22.0)	765 (22.7)
ADMIN3	363 (8.8)	0 (0.0)	363 (8.3)	218 (6.4)
POINT	1,996 (48.6)	0 (0.0)	1,996 (45.9)	1,683 (50.0)
**Total**	4,110	236	4,346	3,372

## Data Records

The dataset aligned to this publication is available for download in.xlsx format at 10.6084/m9.figshare.28879805^13^. The dataset contains 4346 rows and each row represents a unique malaria timeseries. The columns define the source material used to create the rows, geographic and temporal information associated with the timeseries, and descriptors of the type of malaria data that comprises each time series. A full list of the columns contained in the dataset along with descriptions are shown in the Supplemental Information. The geographic coordinates for point-level timeseries are present in the table as latitude and longitude coordinates, and administrative-level data are listed by name and polygon ID. The geometries of the administrative unit polygons matched to the rows in the dataset are available, in shapefile format, at 10.6084/m9.figshare.28879805^13^.

The seasonality timeseries dataset will be periodically updated to incorporate new data, and both this initial and subsequent versions of the dataset will be made available at https://malariaatlas.org/project-resources/predicting-the-effect-of-seasonality-on-malaria-transmission/. The dataset is freely accessible to researchers for unrestricted use with citation of this paper and the repository the data stored within.

## Technical Validation

### Seasonality literature reviewer classifier (SLRC)

**Fig. 4:**
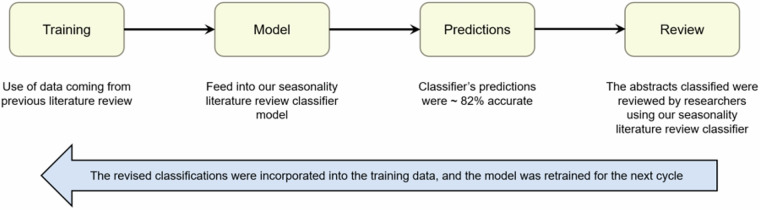
Process used by the seasonality literature review classifier (SLRC).

We developed the SLRC to speed up the literature review process by automatically classifying articles as likely or unlikely to contain information on seasonal patterns of malaria. The SLRC is a machine learning text classification model that uses natural language processing to sort journal articles into categories based on their titles and abstracts (Fig. 4). For this study, the categories were “not relevant for seasonality” and “relevant for seasonality”. More broadly, the approach described below is generalisable and we have successfully applied it for other similar applications. These include developing classifiers to categorise articles as likely to contain extractable malaria prevalence or incidence data.

#### Training data

The SLRC was trained on data collected during a previous malaria seasonality literature review that used *ad hoc* and opportunistic approaches (e.g., identifying suitable timeseries and anecdotes in articles being reviewed for other projects). While the *ad hoc* dataset consisted of several hundred articles, the general consensus is that a minimum of only 10–20 features for each class are required for training naturally language processing models, with more features leading to more accurate results. The titles and abstracts of articles, along with their manual classifications as either not relevant for seasonality or relevant for seasonality, were used as the training data for our SLRC model. The model required that the titles and abstracts first be transformed to convert the text into numerical tokens. This was accomplished by passing them through a count vectoriser and then a term frequency - inverse document frequency transformer from the Scikit-Learn library^14^. The 10,000 most relevant features were selected for both titles and abstracts using *f_classif*, which is class (i.e., a set of methods) within SciKit-Learn (https://scikit-learn.org/stable/modules/generated/sklearn.feature_selection.f_classif.html) that identifies informative features from a sample by computing their ANOVA F-values. The text transformation process was saved as feature transformation pipelines, one each for titles and abstracts, so they could be applied to any new articles fed into the model to receive a prediction. Because the training data retrieved from the previous seasonality literature review contained a high proportion of irrelevant titles and abstracts (i.e., those without seasonality information), only 10% of the articles from the not relevant for seasonality category were randomly selected and used as training data to improve performance of the SLRC.

#### Model

The machine learning model used for classifying the abstracts was built using TensorFlow and is a multi-layer perceptron model. A multi-layer perceptron model is a type of deep, feed forward artificial neural network. The term “deep” means that the model has more than one layer. Specifically, the model consists of an input layer, followed by several alternating and hidden dense and dropout layers, and finally an activation layer. A tanh (hyperbolic tangent) activation function was used within the dense layers. For our purposes softmax was selected as the activation function as it can be used for both simplified and multiclass tasks. To pair with softmax, sparse categorical cross-entropy was selected as the model’s loss function because the classes are mutually exclusive. Alternatively, a binary cross entropy could have been used, but this classifier is just a special case of categorical cross entropy. As such, a binary classifier would have been equivalent, but less flexible were we to add classes in future iterations of this model. Adam was selected as the optimiser^15^, as it is generally considered one of the best optimisers available and is well suited to most problems. A grid search was performed to select the best values for hyperparameters such as the number of hidden layers, the dropout rate, and the dense layers activation function. The training data consisted of 6,469 sources (3,402 type not relevant for seasonality and 3,067 type relevant for seasonality) and were split into a training set, a validation set, and a test set using an 80:10:10 ratio. The out of sample SLRC correctly identified 594 of 647 sources in the testing set, which yields an out-of-sample accuracy of 91.8%. The model sensitivity was 91.5%, as 311 sources contained extractable seasonality information among the 340 sources classified as type seasonality. The model specificity was 92.3%, as determined by the 283 sources correctly deemed to lack seasonality information among the 307 sources classified as type not relevant for seasonality.

#### Predictions

When a source was classified, the model assigned a percentage value to each class. The percentages reflect how sure the model was about the source belonging to a class. Because the classification results consisted of percentages for both classes (e.g. relevant for seasonality: 67%, not relevant of seasonality: 33%), a threshold was necessary for assigning sources to a particular class. To define appropriate thresholds, we plotted receiver operating characteristic curves and calculated the geometric mean for each classification type. We repeated the process for each threshold value, selecting the one with the highest geometric mean for each classification. Based on this analysis, a 60% threshold was used for class not relevant for seasonality and a 40% threshold was used for class relevant for seasonality. The thresholds were determined independently, and thus differ because the source data had a higher number of irrelevant articles compared to those containing seasonality information.

#### Review

To improve the accuracy of the SLRC model, we used an extraction strategy whereby we reviewed articles one year at a time and then retrained the model after each year was complete (Fig. 4). This iterative process was repeated 22 times, once for each year from 2000–2021. To ensure we were not feeding the model its own predictions, we added to its training data only those articles that have been manually reviewed. The model was then retrained on the new, larger training data, checked for accuracy measures, and uploaded for use in the next data collection cycle. The cyclic revision of the SLCR model improved predictive accuracy by increasing the volume of training data available with each revision.

### Data extraction protocol

To ensure data integrity and quality, we developed a data extraction protocol and undertook quality checks. We collected data belonging to one of the four macro categories including prevalence, incidence, entomological, and mortality of malaria. To standardise the metrics within the dataset, their original temporal resolution (e.g. weekly) was reduced to monthly time steps. Data presented in tabular form were ingested into the dataset without modification, while data from plots, graphs, or images were extracted using a semi-automated digital tool (WebPlotDigitizer, version 4.6). The digitisation tool utilised automatic extraction algorithms that enabled the user to extract values following a manual process of selecting the graph type, calibrating the axes, and adding or adjusting data points along the curve^16^. Wherever possible, raw monthly data were extracted for each source, metric, study population age, location, *Anopheles* or *Plasmodium* species, and year. In cases where the raw data were presented in an aggregated form spanning multiple years, we reported it in as a single row. For example, if a 48-month timeseries was collected from 2000 to 2003 but only monthly averages were reported in the paper, the timeseries was included as a single row in the dataset with a start year of 2000 and an end year of 2003. For anecdotal information, we only recorded the peak month(s) of malaria incidence, prevalence, mortality, or entomologic metric provided in the source. Information regarding study location for both anecdotal and empirical rows was retrieved at the lowest administrative unit level (polygon) available, or at point level whereby each record was geolocated with latitude and longitude. In cases where only the name of the administrative unit was provided in the text, the MAP dataset of geometries was searched to identify a matching polygon. Detailed information on the methods used for data gathering are available in Supplemental Information.

Despite using machine learning tools to reduce the amount of time required to identify relevant seasonality sources, most information in the dataset was extracted manually or in a semi-automated way. This might have led to errors, such as inaccurate visual determination of the peak malaria period. Although some timeseries were simple to extract, others required the use of a digitalisation tool to increase the precision and reduce the time required for the data extraction. In other cases, timeseries were not extractable due to the way in which the plot and/or figure were displayed in the paper. The dataset contains a column called “timeseries” to indicate whether the information (i) was extracted from a timeseries, (ii) had an associated timeseries that was not extractable, or (iii) had no associated timeseries. To minimise errors in the malaria timeseries dataset, extractions from plotted data were done twice by independent reviewers. Similarly, to ensure consistency of the definitions for each metric, all data sources were carefully classified, based on the description in the source material, as either incidence, prevalence, mortality, or an entomological metric. This classification step was done twice by independent researchers, and any sources with discrepancies were re-evaluated by project managers. To maintain consistency within the dataset, the data extractors were extensively trained in the thoroughly documented data gathering protocol, and every change in that protocol was documented to maintain transparency in the extraction process. Lastly, after the dataset was assembled, multiple cleaning and validation checks were done to improve the quality and reliability of the data. Data quality and standardisation were focal points for this work to enable comparability between studies. These features of the dataset will provide researchers and decision makers at both national and regional levels with empirical data to support evidence-based strategic planning efforts.

## Supplementary information

Supplemental Information

## Data Availability

The natural language algorithm used to identify potentially relevant articles for subsequent manual review is available at 10.6084/m9.figshare.28879805^13^ and https://malariaatlas.org/project-resources/predicting-the-effect-of-seasonality-on-malaria-transmission/. The article review and dataset assembly were done manually. Malariometric timeseries published only as plots were extracted using WebPlotDigitizer, version 4.6.

## Data availability

The data are available at 10.6084/m9.figshare.28879805^13^ and subsequent revisions will be available at https://malariaatlas.org/project-resources/predicting-the-effect-of-seasonality-on-malaria-transmission/.
