# Supplementary material for: A malaria seasonality dataset for sub-Saharan Africa
Source: Sci Data. 2025 Oct 28;12:1703. doi: 10.1038/s41597-025-05996-5 (PMC12569248; doi:10.1038/s41597-025-05996-5)
Supplement: Supplementary file 1 — Supplemental Information [file 41597_2025_5996_MOESM1_ESM.docx]

**Supplemental Information**

Aim: To create an extensive and accurately representative malaria seasonality database with current and historical information across sub-Saharan Africa. Seasonality is a difficult but important aspect of malaria transmission to capture. We are interested in all sources containing information on malaria seasonality, be it anecdotal evidence or data driven such as timeseries case data.

1. **Scope of Data Search**
   1. Data was collected from the following sources:
      1. Literature (publication year 2000 – 2022)
      2. Malaria Atlas Project Annual Parasite Index (API) database (publication year 1997- 2022)
   2. Geographic extent:
      1. Scope is sub-Saharan Africa where malaria is endemic.
   3. Temporal extent:
      1. 12 months of the year
   4. Types of metrics recorded:
      1. Incidence
      2. Prevalence
      3. Mortality
      4. Entomological data
2. **Data Extraction**
   1. Inclusion criteria:
      1. Data is referring to one of the macro categories: incidence, prevalence, mortality, or entomological data.
      2. Data is reported at the level of an ADMIN unit found in the Malaria Atlas Project (MAP) database or a point associated with a latitude / longitude.
      3. Timeseries data contains 12 consecutive months of data.
      4. Timeseries data collected at a minimum of quarterly intervals (i.e., at least once every three months).
      5. Anecdotal data specifies the peak month(s) of transmission / vector density clearly.
      6. In cases where malaria timeseries plot exists but cannot be extracted, the resulting row will contain information on peak month(s) but no numeric quantities for the months.
   2. Exclusion criteria:
      1. No malaria data contained in the source.
      2. No seasonality data contained in the source.
      3. Anecdotal data discussed in a paper referenced an original source that could be retrieved.
      4. Anecdotal data discussed in a paper referenced an original source that was reported contradictory conclusions or could not be retrieved.
      5. Paper conducted an incomplete year of study, so all 12 months were not accounted for.
      6. Paper was unable to be located, retrieved, or accessed.
      7. Anecdotal data was vague. E.g.: discusses ‘transmission season’ but do not specify the months in which this occurs. Likewise, a peak associated with a ‘rainy season’ but specific months would be excluded.
      8. Timeseries data from graphs that was unable to be retrieved even with the digital tool. E.g., too small or blurred.
      9. Seasonality was only reported for imported cases.
      10. Seasonality was only reported in a location where IRS (indoor residual spraying) or other protective methods (e.g. mass drug administration) were being conducted/ tested.
3. **Data Entry**
   1. **Tracking information:**
      1. ID
         1. A unique integer identifier for each record.
      2. LINK
         1. The URL of the source material (DOI or PubMed ID).
      3. CITATION
         1. The full citation of the data source.
      4. RESOURCE_TYPE
         1. The type of resource the data extracted from.
         2. Must be within the list specified by the data validation rules.
      5. PUBLICATION_YEAR
         1. Year of publication.
         2. Integer from 1997-2022, or ‘unpublished’.
   2. **Participant information:**
      1. STUDY_MONTH_START, STUDY_MONTH_END
         1. The order of months in which the study begins/ ends.
         2. Integer from 1-12.
         3. Anecdotal rows receive an ‘NA’.
      2. STUDY_YEAR_START, STUDY_YEAR_END
         1. The year/s in which the study is completed.
         2. Integer from 1964-2021.
         3. Anecdotal rows receive an ‘NA’.
      3. DATA_OR_ANECDOTE
         1. Is the reported seasonality anecdotal or data driven.
         2. Codified as ‘A’ or ‘D’.
      4. DEMOGRAPHIC
         1. The structure / specific section of the population.
         2. Must be within the list specified by the data validation rules.
      5. AGE_BAND_UNIT, LOWER_AGE_BAND, UPPER_AGE_BAND
         1. The age of the study population.
         2. Must be within the list specified by the data validation rules.
   3. **Site information:**
      1. WHO_REGION
         1. The specifies which of the WHO regions the data is relevant to. Because the dataset it only for Africa, this is limited to AFRO and EMRO.
         2. Must be within the list specified by the data validation rules.
      2. ISO3
         1. Three letter ISO3 country code.
      3. ADMIN0
         1. The country name.
      4. ADMIN1, ADMIN2, ADMIN3
         1. The administration division such as state, province, district, municipality.
         2. Only used for subnational records.
         3. Names matched to specific polygons / geometries for mapping.
      5. SITE_NAME
         1. The precise study location name.
         2. Most relevant for point data.
      6. LOCATION_NOTES
         1. Any additional information describing the site that may be important / informative.
      7. LAT, LONG
         1. The X and Y value of the geographic coordinates in decimal degrees.
         2. Only applicable for point data.
      8. ALTITUDE
         1. Altitude in meters if stated in the source.
      9. ADMIN_LEVEL
         1. The most detailed ADMIN level at which the site can be located.
      10. RURAL_OR_URBAN:
          1. Rural, peri-urban, or urban if stated in the source.
      11. MATCH_ID
          1. The numerical field shared by the polygon shapefile attribute table and the seasonality database. This field supports joining the seasonality table to the shapefile.
          2. Values of -1 are associated with point-level data (i.e., spatial location indicated by the latitude and longitude columns rather than an associated with a polygon).
      12. GEOMETRY_SOURCE
          1. Source polygon dataset associated with the MATCH_IDs
   4. **Seasonality information:**
      1. METRIC
         1. The method of reporting seasonality in the source.
         2. Must be converted to monthly.
      2. METRIC_CAT
         1. The macro-category of the metric extracted.
         2. Codified as “incidence”, “prevalence”, “entomological” or “mortality”.
      3. METRIC_NOTES
         1. Any additional information describing the metric that may be important/ informative.
      4. MOSQUITO_SPECIES
         1. The female Anopheles sp. mosquito vector that carries the Plasmodium sp. parasite.
         2. Only applicable if the metric is vector related and this information is provided.
      5. PLASMODIUM_SPECIES
         1. The focal *Plasmodium* sp. parasite of the study.
         2. Only applicable if the metric is related to malaria transmission or burden and this information is provided.
      6. ORIGINAL_TEMPORAL_RESOLUTION
         1. The original metric collection published in the source data, which includes the defined repeat period.
         2. For data collected at a monthly resolution (i.e., aligned with the monthly structure of this database), this value will match the one listed in the METRIC column.
      7. MONTH_OF_LARGEST_VALUE
         1. The month in which the recorded metric is at its highest.
         2. Can be NA if peak is difficult to differentiate (e.g., on a plot).
      8. JAN_PEAK – DEC_PEAK
         1. Simplified description the peak month(s).
         2. In cases with multiple seasonal peaks, the highest peak is denoted with ‘1’, and secondary, smaller peak(s) are denoted with 0.5.
         3. Must be in [0,1,0.5].
      9. PEAK_PERIOD_OR_HIGHEST_MONTH_RECORDED
         1. Differentiates whether there is a single month of peak transmission, or if multiple months have high values.
         2. Codified as ‘PP’ for peak period, ‘HM’, for highest month, or ‘NA’.
      10. TIMESERIES AVAILABLE
          1. Denotes if timeseries data are available.
          2. Codified as Y, N, or Y_UNABLE_TO_EXTRACT_FROM_PLOT.
      11. JAN - DEC
          1. Numeric values for the metric in each month.
          2. Includes data copied from tables / text and data extracted from plots using digitization tools.
          3. Negative numbers not allowed.
   5. **Source information**
      1. DATABASE_SOURCE
         1. Differentiates whether the data comes from literature review or routine surveillance.
      2. PERSONAL_COMMUNICATION
         1. Was the data for this row obtained via personal communication (e.g., contacting the author of a published paper for the disaggregated data that contributed to their summarized result).
         2. Codified as Y or N.
      3. RIGHT_TO_PUBLISH
         1. Defines whether we have the right to publish the data.
         2. Only publicly available datasets will be released.
         3. Codified as Y or N.

**Supplementary Table 1. Summary of columns in the MAP seasonality database.**

| **1.      Sources of data** | |  |
| --- | --- | --- |
| ID | Unique identifier number for the seasonality record |  |
| LINK | URL to the source DOI or PubMed ID |  |
| CITATION | The formal citation of the data source |  |
| RESOURCE_TYPE | The type of source |  |
| PUBLICATION_YEAR | Year of publication |  |
| DATABASE_SOURCE | Sources retrieved by the new literature review or the MAP routine surveillance database |  |
| PERSONAL_COMMUNICATION | Were the data obtained via personal communication |  |
| RIGHT_TO_PUBLISH | Do we have the right to publish the data |  |
|  |  |  |
| **2.      Dates of data collection** | |  |
| STUDY_MONTH_START | Month of the study start |  |
| STUDY_YEAR_START | Year of the study start |  |
| STUDY_MONTH_END | Month of the study end |  |
| STUDY_YEAR_END | Year of the study end |  |
| DATA_OR_ANECDOTE | Is the reported seasonality anecdotal or data driven. If data contains extractable data, timeseries data extraction procedures were followed. |  |
|  |  |  |
| **3.      Demographic information** | |  |
| DEMOGRAPHIC | Highlight only if a specific group of the population is studied. Otherwise, “all”. |  |
| AGE_BAND_UNIT | Age band unit of the population under study |  |
| LOWER_AGE_BAND | Lower age limit of the population under study |  |
| UPPER_AGE_BAND | Upper age limit of the population under study |  |
|  |  |  |
| **4.      Geographical location** | |  |
| WHO_region | WHO Region |  |
| ISO | ISO of the country (3 digits) |  |
| ADMIN_LEVEL | One of POINT, ADMIN0, ADMIN1, ADMIN2, or ADMIN3 |  |
| ADMIN0 | The country of study |  |
| ADMIN1 | Optional, the first (level) administration division such as states, provinces, or regions. |  |
| ADMIN2 | Optional, the second (level) administration division such as districts or counties. |  |
| ADMIN3 | Optional, the third (level) administration division such as municipalities or communes. |  |
| SITE_NAME | Optional, the exact name of the place where the source said the study was carried out |  |
| LOCATION_NOTES | Any other information that may be useful when trying to geo-position a site |  |
| LAT | Required if sites name are given and data are points rather than admin units. The Y value of the geographic coordinates is in decimal degree |  |
| LONG | Required if sites name are given and data are points rather than admin units. The X value of the geographic coordinates is in decimal degree |  |
| ALTITUDE | if reported by the source - in meters |  |
| RURAL_OR_URBAN | If rural, urban, or peri-urban is clearly reported by the source |  |
| MATCH_ID | Numerical identifier of the associated geometry for polygon data |  |
| GEOMETRY_SOURCE | The database from which the associated polygon in the shapefile was copied |  |
|  |  |  |
| **5.      Malaria metric captured within the data** | |  |
| METRIC | The malariometric data constituting the timeseries after conversion to monthly data |  |
| METRIC_CAT | Metric macro category |  |
| METRIC_NOTES | Any other information that may be useful related to the metric |  |
| MOSQUITO_SPECIES | If the metric is vector related and this information is given |  |
| PLASMODIUM_SPECIES | If the information is given |  |
|  |  |  |
| **6.      Measured malaria-relevant metrics at a monthly resolution** | |  |
| ORIGINAL_TEMPORAL_RESOLUTION | The malariometric data constituting the timeseries in its original temporal resolution |  |
| MONTH_OF_LARGEST_VALUE | When values are reported for each month, it gives you the month with the highest value. Otherwise, from article |  |
| JAN_PEAK– DEC_PEAK | If the peak month is January - December |  |
| PEAK_PERIOD_OR_HIGHEST_MONTH_RECORDED | Identify whether the peak period or the highest month is recorded |  |
| TIMESERIES_AVAILABLE | Does the source contain timeseries? |  |
| JAN- DEC | Raw values from timeseries for January to December |  |
| WMR_REPORTING_COMPLETENESS | Year-matched, national-level public-sector data reporting completeness (from the World Malaria Report) that may provide users with additional context for interpreting timeseries from routine surveillance. |  |
|  |  |  |
